# *Slice’N’Dice*: maximizing the value of predicted models for structural biologists

**DOI:** 10.1107/S2059798325001251

**Published:** 2025-02-20

**Authors:** Adam J. Simpkin, Luc G. Elliot, Agnel Praveen Joseph, Tom Burnley, Kyle Stevenson, Filomeno Sánchez Rodríguez, Maria Fando, Eugene Krissinel, Stuart McNicholas, Daniel J. Rigden, Ronan M. Keegan

**Affiliations:** a Institute of Structural, Molecular and Integrative Biology University of Liverpool LiverpoolL69 7ZB United Kingdom; b UKRI–STFC Rutherford Appleton Laboratory Research Complex at Harwell DidcotOX11 0FA United Kingdom; c York Structural Biology Laboratory, Department of Chemistry University of York York United Kingdom; European Bioinformatics Institute, United Kingdom

**Keywords:** structure determination, cryo-EM, X-ray crystallography, molecular replacement, structure prediction

## Abstract

With the advent of next-generation modelling methods, such as *AlphaFold*2, structural biologists are increasingly using predicted structures to obtain structure solutions via molecular replacement (MR) or model fitting in single-particle cryogenic sample electron microscopy (cryoEM). Differences between the domain–domain orientations represented in a predicted model and a crystal structure are often a key limitation when using predicted models. *Slice’N’Dice* is a software package designed to address this issue by first slicing models into distinct structural units and then automatically placing the slices using either *Phaser*, *MOLREP* or *PowerFit*. The slicing step can use the *AlphaFold* predicted aligned error (PAE) or can operate via a variety of C^α^-atom-based clustering algorithms, extending the applicability to structures of any origin. The number of splits can either be selected by the user or determined automatically. *Slice’N’Dice* is available for both MR and automated map fitting in the *CCP*4 and *CCP-EM* software suites.

## Abstract 2

Recent advancements in computational structural modelling have provided structural biologists with access to high-quality predictions that can be used to aid experimental structure determination. *Slice’N’Dice* is a new application from *CCP*4 that is designed to help with the preparation of these predictions for the structure-solution process, particularly where the prediction needs to be split in order to better match the experimental structure.

## 1. Introduction

In macromolecular X-ray crystallography (MX), molecular replacement (MR) remains the dominant method for solving the phase problem, with 92.8% of the crystal structures deposited in the Protein Data Bank (PDB; Burley *et al.*, 2021 ▸) between November 2023 and October 2024 having been solved by MR. The emergence of next-generation predicted models has wide-reaching implications for MX, with MR being a key application. The availability of sufficiently close homologues with experimentally determined structures has always been a limitation in MR, one which is largely solved by the highly accurate models produced by next-generation modelling methods such as *AlphaFold*2 (Jumper *et al.*, 2021 ▸), *RosettaFold* (Baek *et al.*, 2021 ▸) and *ESMFold* (Lin *et al.*, 2023 ▸).

In MX, studies (McCoy *et al.*, 2022 ▸; Terwilliger *et al.*, 2024 ▸; Keegan *et al.*, 2024 ▸) have shown that using high-quality predictions as search models in MR can solve the vast majority of cases, even where the original structure determination employed experimental phasing. To facilitate this, some preprocessing of the predicted model is often required for success in MR and the same is true for cryoEM map fitting. The quality of the predicted model can vary across the target sequence, with some regions being inaccurately predicted. *AlphaFold*2, *RosettaFold* and *ESMFold* each provide predicted quality scores on a per-residue basis that can be used to guide the removal of any residues that are likely to have been inaccurately modelled. *AlphaFold*2 and *ESMFold* give the predicted local distance difference test (pLDDT) score (Jumper *et al.*, 2021 ▸), a per-residue estimate of its confidence on a scale from 0 to 100 and 0 to 1, respectively, where higher values correspond to higher confidence. *RosettaFold* gives an estimated root-mean-square deviation (r.m.s.d.), a per-residue estimate of the r.m.s.d. to the true structure, where lower values correspond to higher confidence. The methods store this information in the *B* factor column of their output PDB files.

While local confidence scores work well for estimating the reliability of individual residues, they are unable to indicate global inaccuracies in the model such as those caused by inter-domain conformational changes. To address this problem, *AlphaFold*2 provides a predicted aligned error (PAE; Varadi *et al.*, 2022 ▸) matrix. The PAE shows the expected error in the distances between residues. Low PAE values signify high confidence and, when sustained over a range of residues, often correspond to well defined structural domains, while high PAE values indicate greater uncertainty and are typically found in regions between domains or in more flexible parts of the protein. The PAE can therefore be used to assess the reliability of a predicted inter-domain orientation.

Another important step for MX is the conversion of the pLDDT/r.m.s.d. values into pseudo-*B* factors. When using PDB-derived search models, *B* factors are used for weighting search models in *Phaser* (McCoy *et al.*, 2007 ▸), and therefore the use of pseudo-*B* factors can improve the performance of the models in MR (Croll *et al.*, 2019 ▸; Oeffner *et al.*, 2022 ▸).

Here, we present *Slice’N’Dice*, an automated pipeline to efficiently process and deploy deep-learning-based structure predictions in both the MX and cryoEM fields. It first processes predicted models by removing low-confidence regions and converting confidence scores into pseudo-*B* factors. It then slices predicted models into distinct structural units which can be placed in an automated fashion. With MR, a strategy is employed which either provides *Phaser* (McCoy *et al.*, 2007 ▸) with all of the slices or attempts to place the slices individually before combining any placements that are deemed to be successful (hybrid mode), while in cryoEM map fitting a novel machine-learning model is used to guide the sequential acceptance of placed structural units. Taken together, these pipelines allow *Slice’N’Dice* to maximize the effectiveness of predicted models in both MR and EM map fitting.

## 2. Methods

*Slice’N’Dice* is a combination of two steps: ‘*Slice*’, which breaks models up into distinct structural units, and ‘*Dice*’, a step that was originally developed to perform automated MR on the split models (named as a nod to the maximum-likelihood methods in *Phaser*) but that now also encompasses map fitting for cryoEM.

### 2.1. Slice

#### 2.1.1. Clustering

**Figure 1:**
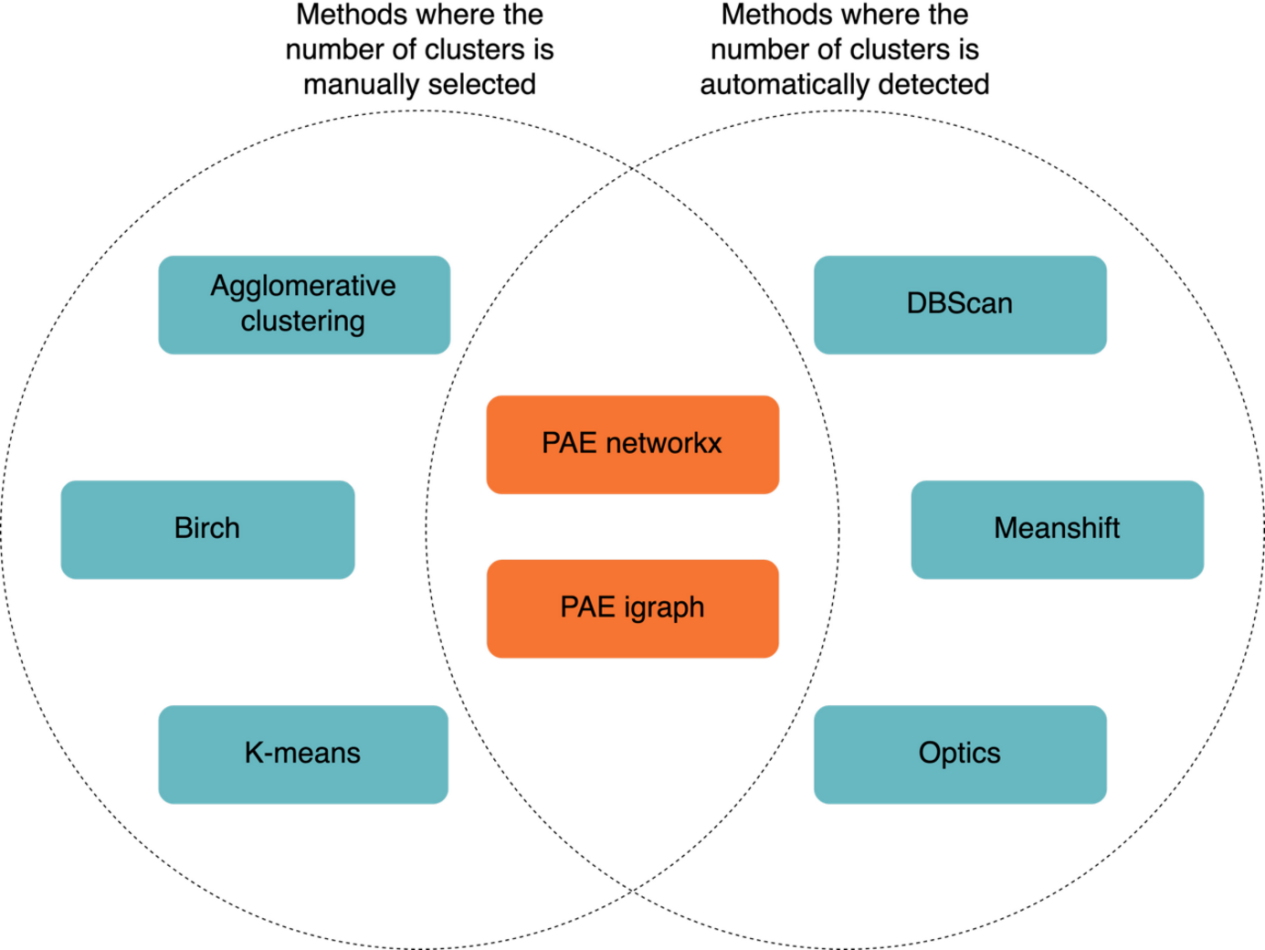
Venn diagram showing the various clustering methods used to split models into distinct structural units and included in *Slice’N’Dice*. Shown in teal are clustering methods included in *scikit-learn* that cluster based on C^α^-atom coordinates. Shown in orange are clustering methods included in *cctbx* that cluster based on the predicted aligned error (PAE) from *AlphaFold*2. On the left are all of the clustering methods that require the number of clusters to be specified and on the right are clustering methods that automatically determine the number of clusters. The *cctbx* PAE methods automatically identify clusters: however, if a user defines a maximum number of splits, *Slice’N’Dice* performs an additional step to merge the closest clusters until the number of splits is less than or equal to the maximum number of splits.

Clustering algorithms are used to detect distinct structural units within a predicted model. *Slice’N’Dice* provides eight clustering methods for users to choose from (Fig. 1 ▸). Six clustering methods, coloured teal in the figure, are used from the *scikit-learn* machine-learning library (version 1.0.2; Garreta & Moncecchi, 2013 ▸). These exploit the proximity of atoms in domains to clusters based on the coordinates of the C^α^ atoms. Two other PAE-based methods are also provided from the *Computational Crystallography Toolbox* library (*cctbx*; Grosse-Kunstleve *et al.*, 2002 ▸). Both cluster on the PAE output from *AlphaFold*2 (Oeffner *et al.*, 2022 ▸). Based on preliminary data, the *BIRCH* algorithm (*Balanced Iterative Reducing and Clustering using Hierarchies*; Zhang *et al.*, 1996 ▸) has been found to be the most effective and is the current default in *Slice’N’Dice*.

**Figure 2:**
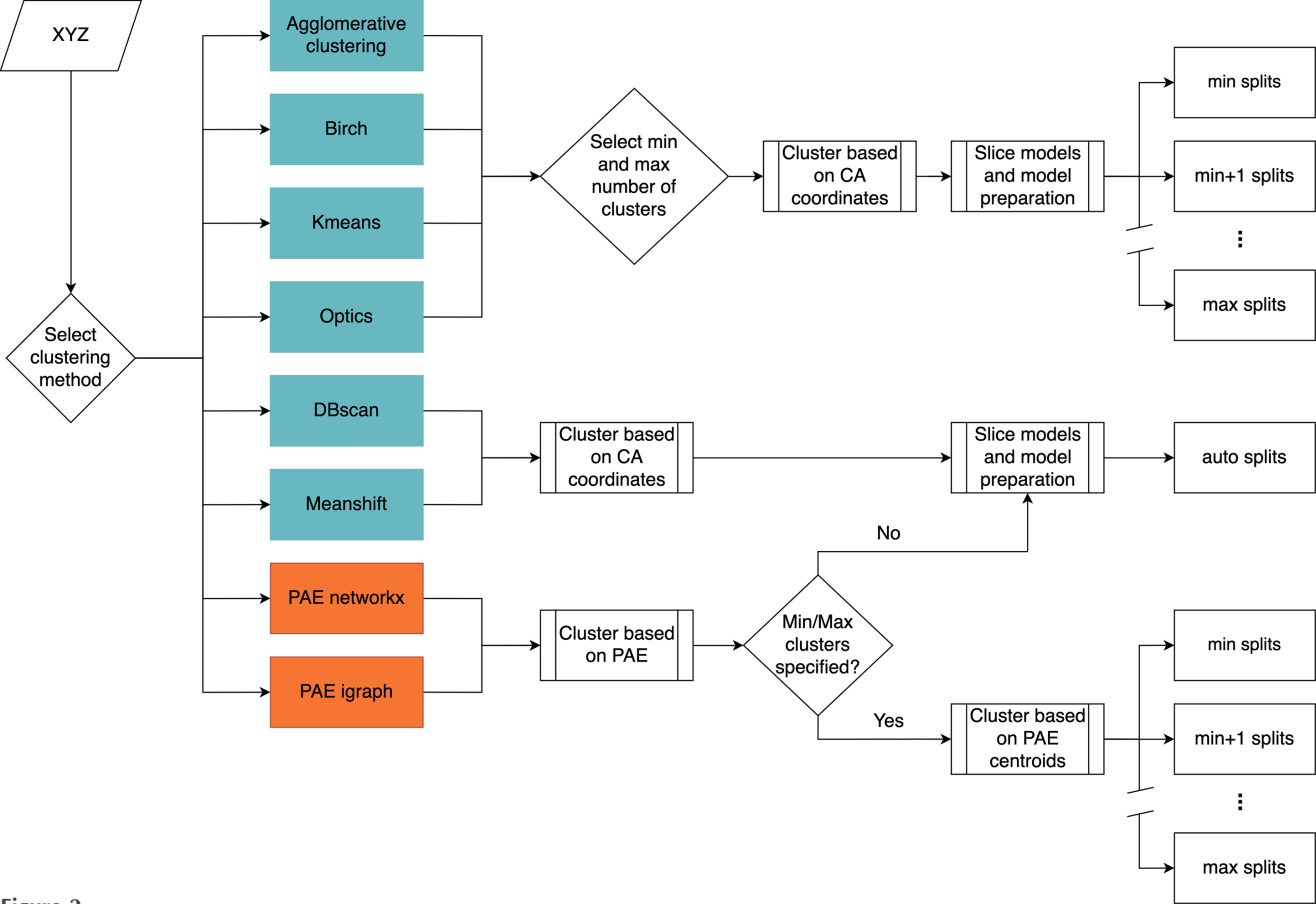
Flowchart showing the model-slicing process. *Scikit-learn* methods are shown in teal and *cctbx* methods are shown in orange.

The clustering methods can be subdivided further into those methods which automatically determine the number of clusters to produce and those methods which require users to manually specify the number of clusters to produce. The two PAE-based methods produce automatically determined structural units, but *Slice’N’Dice* allows these to be combined where a user has specified a smaller number of slices by calculating the centroid for each cluster and clustering these centroids using the agglomerative clustering algorithm. For those methods where the number of clusters needs to be or can be specified, users can set the minimum and maximum number of splits to be made. This allows *Slice’N’Dice* to test a range of different splits (Fig. 2 ▸).

In some cases, particularly when fitting to a cryoEM map, target structures can be very large and may require separate predictions of component parts. To handle this scenario, the program can be given a list of predicted models as input. The number of times that each individual input model is split can also be specified when using manual clustering options.

#### 2.1.2. Model truncation and *B* factor treatment

The type of score contained in the *B* factor column of a model coordinate file (for example pLDDT for *AlphaFold*2, r.m.s.d. for *RosettaFold* and fractional pLDDT for *ESMFold*) can be specified by the user. Predicted models often require some form of truncation to succeed in MR. Low-confidence residues in the predicted model are unlikely to have the same conformation in a crystal or cryoEM structure. *Slice’N’Dice* manipulates the *B* factor column data from a predicted model in two ways.

(i) Per-residue quality scores contained in the *B* factor column of the predicted models (for example pLDDT) are used to direct the truncation of the predicted model. Residues that score below a specified threshold are removed from the model.
(ii) The per-residue quality scores are converted to pseudo-*B* factors using the methods described in Simpkin *et al.* (2022 ▸) and Croll *et al.* (2019 ▸). *Phaser* makes use of atomic *B* factors in its maximum-likelihood method, helping to weight their contribution in the MR search.

For *AlphaFold*2 models, the default pLDDT threshold is 70 and for *RosettaFold* models the default RMS threshold is 1.75. Both values can be set by the user. If using models that have already undergone a pseudo-*B* factor conversion, the conversion step can be skipped. To enable the truncation of poorly predicted regions in this scenario, the given pLDDT threshold value is converted into a pseudo-*B* factor and any residues scoring above this value are removed.

### 2.2. Dice

The second part of the *Slice’N’Dice* pipeline, ‘*Dice*’, performs molecular replacement or map fitting using the individual slices produced by ‘*Slice*’.

#### 2.2.1. MX *Dice*

**Figure 3:**
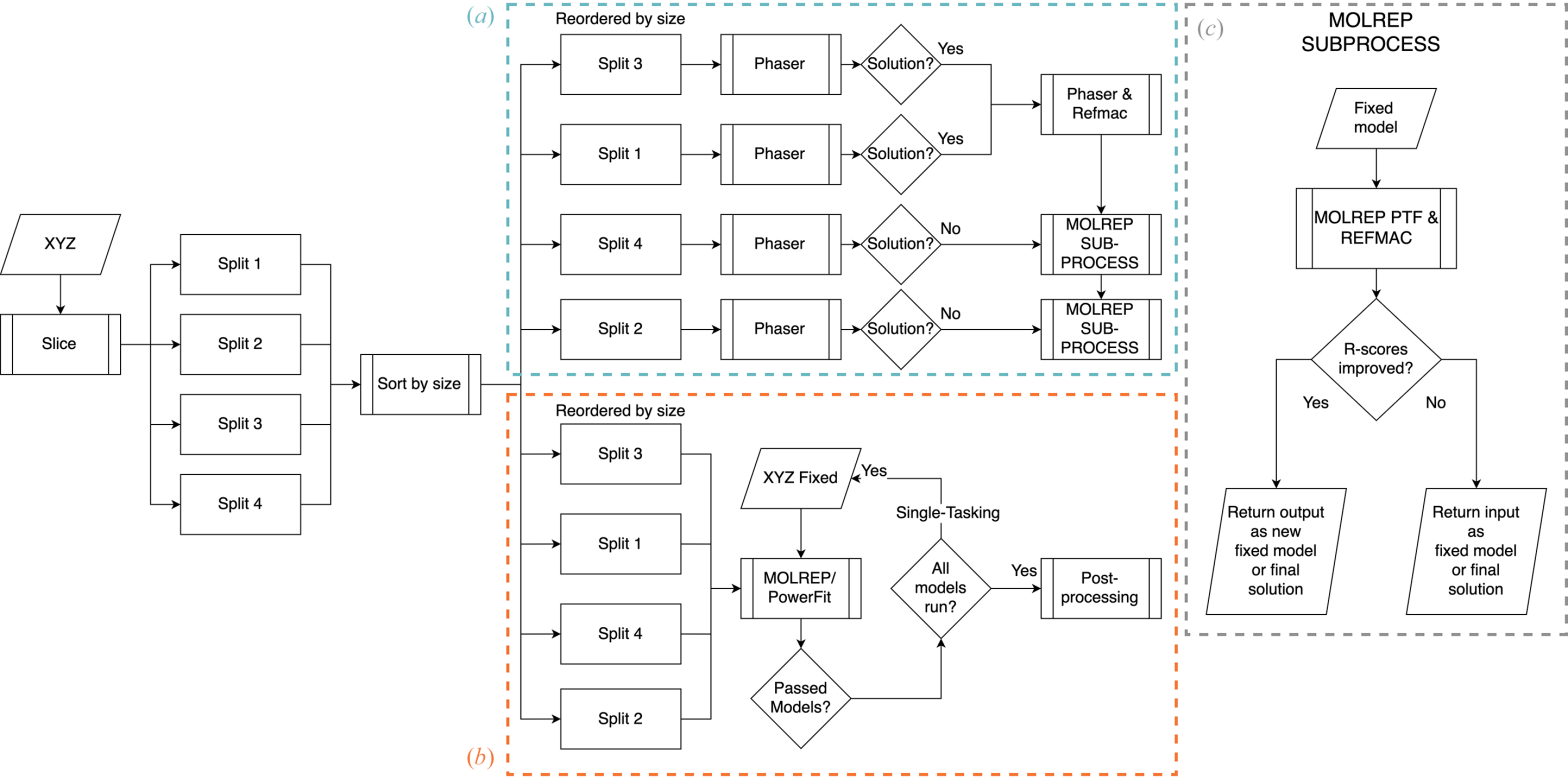
Flowchart showing (*a*) the *Slice’N’Dice* hybrid MR mode, where *Phaser* jobs are ordered by slice size and run in order if a single processor is specified or run in parallel if multiple processors are specified. Any solutions found in this initial *Phaser* step are combined and used as a fixed model that is input into the *MOLREP* subprocess [detailed in (*c*)]. (*b*) The *Slice’N’Dice* EM pipeline. (*c*) The *MOLREP* PTF step where we attempt to place additional slices from a fixed input model. If the *R* scores improve the output is either set as a fixed model for subsequent slices or returned as our final MR solution.

In the default mode, *Dice* provides all of the slices to *Phaser* (McCoy *et al.*, 2007 ▸) simultaneously to automatically place as many slices as possible. This strategy works in the vast majority of cases, but in some situations smaller parts of the sliced model can be difficult to place through standard MR. To aid with their placement we incorporated an additional search step making use of a phased translation function (PTF; Read & Schierbeek, 1988 ▸). This uses the phases generated from those slices that have already been successfully placed by *Phaser* (achieving a per-slice LLG of ≥60) to improve the chances of placing smaller search models. The current implementation of *Slice’N’Dice* makes use of *MOLREP* (Vagin & Teplyakov, 2010 ▸) to perform this step, but it could also be performed using *Phaser*. Specifically, we make use of the SAPTF (spherically averaged phased translation function) implementation from *MOLREP* where the position of the centre of mass of a search model is found prior to determination of its orientation. The orientation is subsequently found by a phased rotation function (Vagin & Isupov, 2001 ▸). After each *MOLREP* job, *REFMAC*5 (Murshudov *et al.*, 2011 ▸) is used to assess whether the placed slice has improved the solution. Fig. 3 ▸(*a*) shows the decision-making process used in the hybrid mode.

#### 2.2.2. CryoEM *Dice*

When provided with a cryoEM density reconstruction (map), the *Slice’N’Dice* EM pipeline makes use of two automatic map-fitting programs: *MOLREP* and *PowerFit* (van Zundert & Bonvin, 2015 ▸). *MOLREP* runs on a single core, which means that multiple splits can be docked into a map file simultaneously, providing an efficient form of map fitting for a CPU-based workstation. Alternatively, *Powerfit* performs an exhaustive rotational and translational search across the map. This has a high computational cost on CPU-based workstations, but these computations can be offloaded to the GPU, reducing the processing time drastically. *MOLREP* is run by default and is distributed as part of the *CCP*4 and *CCP-EM* software suites. *PowerFit* needs to be installed as an additional dependency. This can be performed using *package-ccpem*2 (https://gitlab.com/ccpem/package-ccpem2). Fig. 3 ▸(*b*) illustrates the overall *Dice* pipeline for EM, although slight differences exist between the methodology depending on how the map-fitting programs utilize the hardware. *MOLREP*can be run in parallel and the top, non-overlapping, models that pass a machine-learning classifier (Section 2.2.2.1) are returned. *PowerFit* will run sequentially using the previous fitted model (assuming that it has passed the checking process) as a fixed model.

##### 2.2.2.1. Map–model binary classifier

Assessing the suitability of the map-fitted models can be accomplished through a trained eye and validation metrics; however, automating this process presents a significant challenge. To tackle this issue, a machine-learning approach was employed. The map–model fitting scores ultimately included in the training data for the machine-learning classifier were Fourier shell correlation average (FSCavg), mutual information (MI), cross correlation (CC) and segment-based Manders’ overlap coefficient (SMOC). Also included are overlap map and overlap model scores. These give the classifier additional information about the relative size of the map/model. Additional information on the classifier training, the calculation of the map–model scores and hyperparameter optimization can be found in the supporting information.

A training data set of approximately 14 000 rows of scores is generated as input for the classifier. Typically, a test–train split is performed for training purposes. During preliminary training of the data, overfitting was a significant concern, given that multiple protein models can be trained on a single map. To mitigate this effect, a separate, smaller training data set was generated so that the classifier could be tested against maps that it has not encountered. The data set was balanced using undersampling, and any noise from the score-generation process was eliminated.

Various machine-learning binary-classification models were tested as part of the training process; all of these are available through the *scikit-learn* Python package (Pedregosa *et al.*, 2011 ▸). The models tested were support vector classifier, *k*-nearest neighbours classifier, random forest classifier, extra trees classifier and stochastic gradient descent (SGD) classifier. Each of these models was accessible through *scikit-learn*. SGD is not inherently a classifier but implements different classifiers and uses the SGD algorithm for optimization. The efficacy of the model depends on the chosen loss. Out of these classifiers, SGD was chosen for the task due to its preliminary aptitude and reduced computational time for training, which greatly sped up the hyperparameter-testing process.

To fine-tune the model, the *scikit-learn* class Randomized­SearchCV was utilized (Pedregosa *et al.*, 2011 ▸), employing a range of different hyperparameters to optimize the accuracy scoring function. Choosing an alternative scoring metric resulted in the classifier heavily favouring one class to maximize the score, whereas optimizing for accuracy led to more balanced predictions. See Table 1 ▸ for the hyperparameters, their search spaces and the selected value used for the final training of the classifier. Various loss functions were tested during the hyperparameter stage despite choosing log loss for the final classifier round. This ensured a probability score which is used in the *Slice’N’Dice* clash checker (see below). When selecting log loss, the SGD classifier employs logistic regression.

##### 2.2.2.2. Clash checker

During map fitting, multiple models can be placed in a way in which they overlap with one another. Each slice is run with *MOLREP* concurrently across the entire search space in the map, and therefore the outputs can overlap. Issues can also arise with *PowerFit* when it places models in close proximity. To mitigate this, if two models share the same bounding box, a clash checker is run to determine whether and to what extent they overlap.

To prevent two models occupying the same space (overlapping), a ball-tree algorithm is used. The ball tree is a data structure used for efficient nearest-neighbour searches in high-dimensional spaces by recursively partitioning data points into nested hyperspheres or balls (Omohundro, 1989 ▸). In our case, the data points are the atom model coordinates. A ball tree is able to make efficient comparisons of distances between itself and another ball tree, making it more resilient to larger input sizes (here larger atomic models). The ball tree is calculated using the *scikit-learn* Python package (Pedregosa *et al.*, 2011 ▸). Currently, the overlap check examines atoms within a distance threshold of 3.8 Å. This threshold is based on the average r.m.s.d. of the distances between two continuous C^α^ atoms in an atomic model (Chakraborty *et al.*, 2013 ▸); the rationale is that the C atoms on the opposing protein structure should fall outside this range. If more than 5% of atoms in the shorter model extend beyond this threshold, the models are considered to be overlapping. The 5% threshold was chosen to allow for small overlapping regions that might potentially be resolved later without obstructing the discovery of a global solution. If a clash is found, the protein model with the higher classifier-calculated probability value is chosen and the other is discarded.

### 2.3. Assessing the results

#### 2.3.1. MX

An all-atom r.m.s.d. (with outlier rejection) was calculated between the target and the model before and after *Slice’N’Dice* using *PyMOL* (https://www.pymol.org/) to assess the improvement in the overall alignment achieved by slicing the model. MR in *Phaser* was considered to be successful when the log-likelihood gain (LLG) improved by 60 or more and the translation-function *Z*-score (TFZ) was ≥8 for each placed slice (Oeffner *et al.*, 2018 ▸). By default *Slice’N’Dice* also performs ten cycles of jelly-body refinement (increased to 100 as of version 0.1.1) using *REFMAC*5 (Murshudov *et al.*, 2011 ▸), with *R* scores of ≤0.45 considered to be indicative of a solution. To verify any solutions, *phenix.get_cc_mtz_pdb* (Liebschner *et al.*, 2019 ▸) was used to calculate the map correlation coefficient (mapCC) score against the deposited structure, with a global mapCC score ≥0.25 being considered to be a success.

#### 2.3.2. CryoEM

A correlation coefficient (CC) was calculated using the *ChimeraX* fitmap function with model shift and rotation deactivated to restrain the current position of the model in the map for scoring (Pettersen *et al.*, 2021 ▸). Unlike CC for MX, CC for cryoEM does not have a defined threshold for a solution, being more helpful for comparisons of alternative possible solutions. Nevertheless, Supplementary Table S3 provides an insight into the distribution of CC scores of EMDB-deposited cryoEM maps and their corresponding protein models. From this distribution, a CC greater than 0.507 and 0.559 (Supplementary Table S3) in the resolution ranges 4.5–6 Å and >6 Å, respectively, is likely to indicate a good fit; anything less than 0.408 and 0.4345, respectively, is likely to suggest a misfit.

## 3. Results

### 3.1. Results overview

*Slice’N’Dice* can enable a more effective use of structure predictions in MR. In this way, some cases that would otherwise be difficult or intractable can readily be solved. Here, we show a number of examples, deposited after the release of *AlphaFold*2, that highlight the ways in which *Slice’N’Dice* can maximize the effectiveness of predicted models in MR and in cryoEM. The structure predictions used in the MX testing were generated using *AlphaFold*2 (Jumper *et al.*, 2021 ▸), while the structure predictions used in the cryoEM testing were generated using *ColabFold* (Mirdita *et al.*, 2022 ▸).

### 3.2. MX examples

#### 3.2.1. Example 1: PDB entry 7oa7

**Figure 4:**
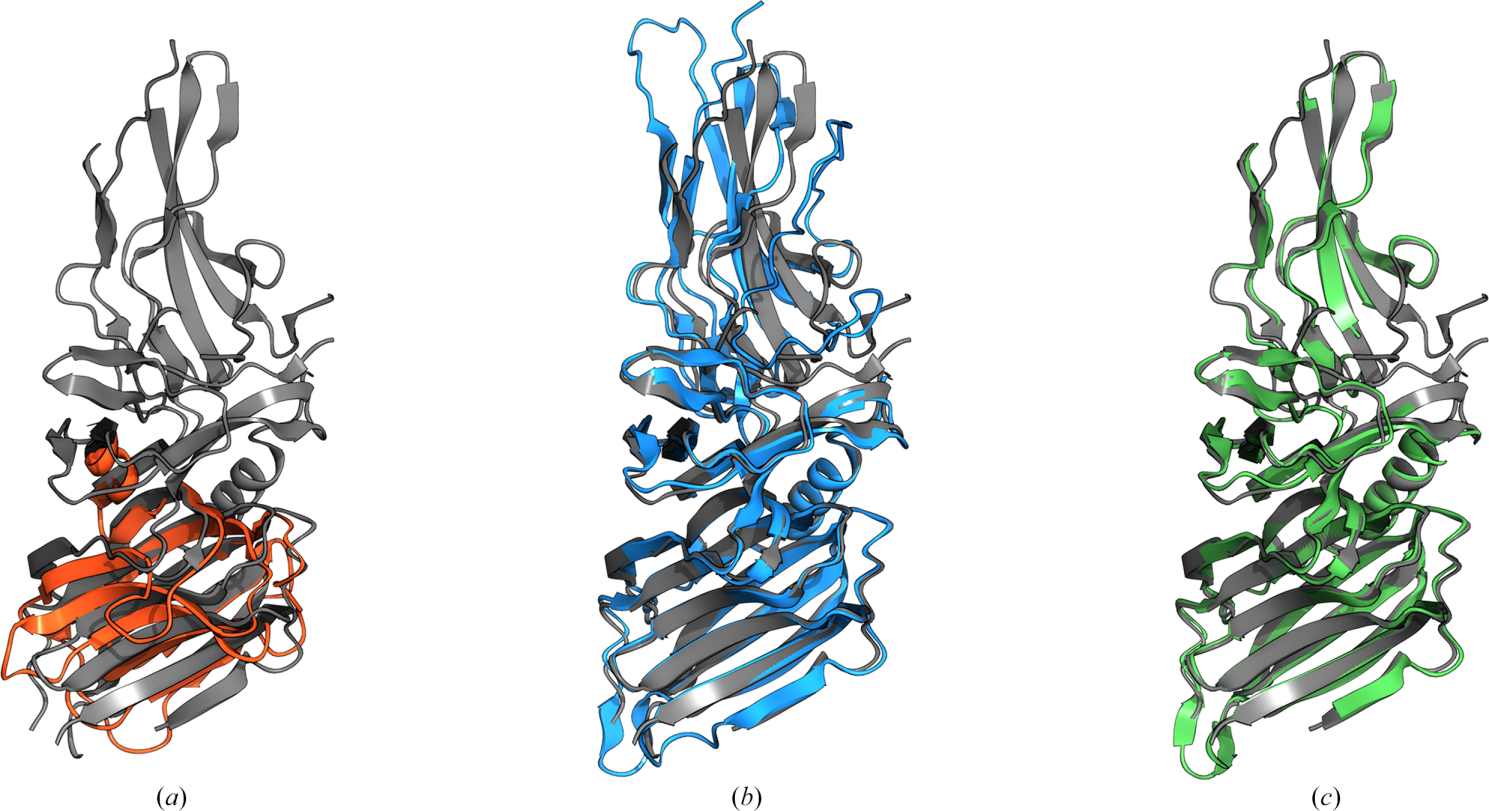
(*a*) The closest match in the PDB to the target structure, PDB entry 3asi (orange, r.m.s.d. 10.88 Å), superimposed on the crystal structure of PDB entry 7oa7 (grey). (*b*) An *AlphaFold*2 model of the target (blue, r.m.s.d. 1.56 Å) superimposed on the crystal structure of PDB entry 7oa7 (grey). (*c*) The *AlphaFold*2 model after slicing and MR with *Slice’N’Dice* (green, r.m.s.d. 0.26 Å, global mapCC 0.7) shown against the crystal structure of PDB entry 7oa7 (grey). This figure was made using *Moorhen* (https://moorhen.org/).

PDB entry 7oa7 is a crystal structure of a PilC minor pilin solved by single-wavelength anomalous dispersion (SAD). At the time of its release, the closest hit in the PDB (PDB entry 3asi) had only 12% sequence identity to the target and was insufficiently similar to succeed as an MR search model (Fig. 4 ▸*a*). A model made by *AlphaFold*2 had very good predicted quality overall (average pLDDT 85.61) but was unable to solve the structure since *AlphaFold*2 modelled a different conformation between the two domains (Fig. 4 ▸*b*). By using the *BIRCH* algorithm in *Slice’N’Dice* to split the structure into two, the structure can readily be solved by MR with a final LLG of 1339 and a global mapCC of 0.7 (Table 2 ▸, Fig. 4 ▸*c*). This structure could also be solved using the PAE *networkx* algorithm (Hagberg *et al.*, 2008 ▸; Oeffner *et al.*, 2022 ▸) with the maximum number of splits set to two (Table 2 ▸). *BIRCH* and PAE *networkx* identified slightly different domain boundaries (Supplementary Fig. S1), and whilst *BIRCH* seemed to work slightly better in this case, both methods could be refined to the same point.

#### 3.2.2. Example 2: PDB entry 7rb4

**Figure 5:**
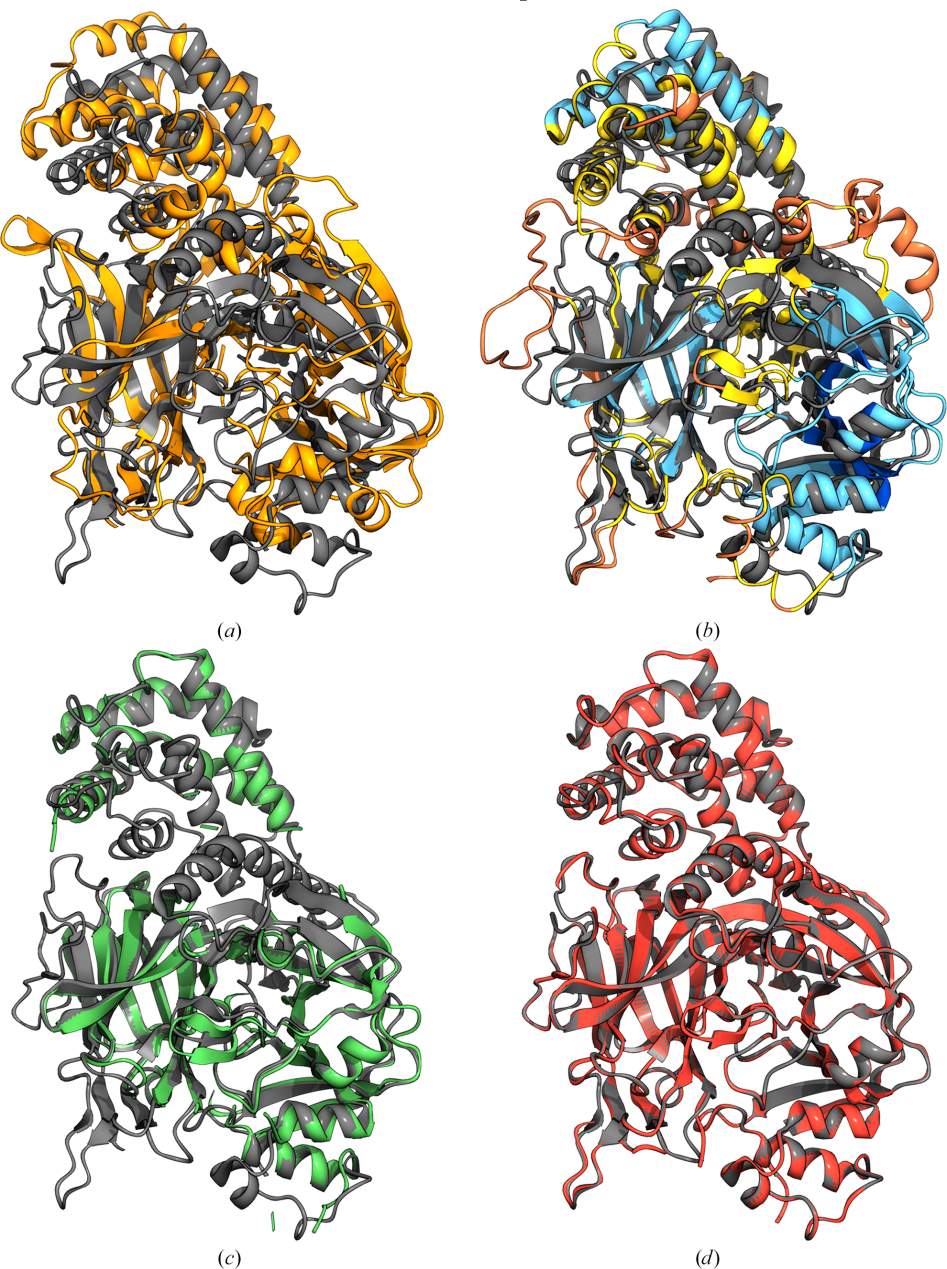
(*a*) The closest match in the PDB to the target structure, PDB entry 1f0l (orange, r.m.s.d. 5.74 Å), superimposed on the crystal structure of PDB entry 7rb4 (grey). (*b*) An *AlphaFold*2 model of the target coloured on a scale of orange to blue, where orange indicates a low pLDDT score (≤50) and blue indicates a high pLDDT score (≥90), superimposed (r.m.s.d. 3.19 Å) on the crystal structure of PDB entry 7rb4 (grey). (*c*) The *AlphaFold*2 model after preprocessing, slicing and MR with *Slice’N’Dice* (green, r.m.s.d. 0.32 Å), shown against the crystal structure of PDB entry 7rb4 (grey). (*d*) The placed *AlphaFold*2 model after 20 cycles of model building with *Buccaneer* (red, r.m.s.d. 0.14 Å, global mapCC 0.84) shown against the crystal structure of PDB entry 7rb4 (grey). This figure was made using *Moorhen*.

PDB entry 7rb4 is a crystal structure of peptono toxin solved by SAD. The closest hit in the PDB (PDB entry 1f0l) had only 26% sequence identity to the target and was insufficiently similar to work in MR, even when split with *Slice’N’Dice* (Fig. 5 ▸*a*). A model made by *AlphaFold*2 was poor quality overall (average pLDDT 61.02; Fig. 5 ▸*b*). Indeed, simply splitting the model with default *Slice’N’Dice* failed to lead to a structure solution. Nonetheless, the combination of the removal of residues below a relaxed pLDDT threshold of 50 with splitting the model into three units, steps implemented together in *Slice’N’Dice*, led to structure solution (LLG 114, *R* factor 0.44, *R*_free_ 0.47, mapCC 0.59; Fig. 5 ▸*c*). This solution could be significantly improved by running 20 cycles of *Buccaneer* (Cowtan, 2006 ▸), which increased the percentage of modelled residues from 34 to 74 (completeness by residues 0.74, *R* factor 0.23, *R*_free_ 0.30, global mapCC 0.84; Fig. 5 ▸*d*).

#### 3.2.3. Example 3: PDB entry 7b9c

**Figure 6:**
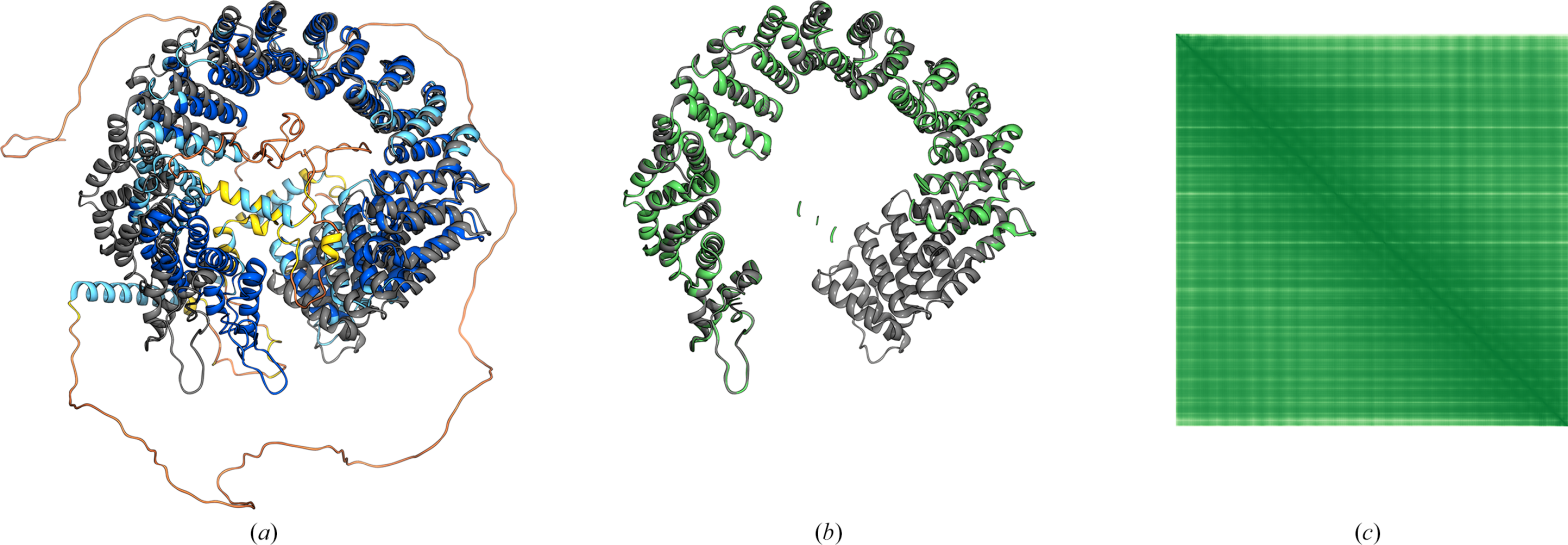
(*a*) O75533, a model from the AlphaFold Protein Structure Database, coloured on a scale of orange to blue, where orange indicates a low pLDDT score (≤50) and blue indicates a high pLDDT score (≥90), aligned (r.m.s.d. 3.95 Å) with the SF3B core (chain *C*) from PDB entry 7b9c (grey). (*b*) O75533 after preprocessing, slicing and MR with *Slice’N’Dice* (green, r.m.s.d. 0.3 Å, global mapCC 0.47), shown against the SF3B core (chain *C*) from 7b9c (grey). This figure was made using *Moorhen*. (*c*) An EPE plot from *AlphaFold3* for O75533.

PDB entry 7b9c is a crystal structure of a minimal splicing factor 3B (SF3B) core in complex with spliceostatin A solved by MR using PDB entries 5ife and 6en4 as search models. Despite highly similar homologues in the PDB, a model of SF3B subunit 1 deposited in the EBI AlphaFold Protein Structure Database (Varadi *et al.*, 2022 ▸; UniProt ID O75533) was insufficiently similar to the target protein to succeed in MR (Fig. 6 ▸*a*). The HEAT repeat region of SF3B is confidently predicted by *AlphaFold*2, but has been predicted to adopt a much tighter conformation than the crystal structure. Without reference to the solved structure, it would be unclear to the experimentalist where the model should be split manually in order for it to succeed in MR. However, the *Slice’N’Dice* automated slicing procedure was able to successfully slice the model into four structural units, of which three could be placed by MR (LLG 310) and used to solve the structure (Fig. 6 ▸*b*). The refinement scores were a little high (*R* factor 0.48, *R*_free_ 0.51, local mapCC 0.8) due to the fact that the SF3B subunit 1 domain made up only 43.8% of the total scattering content. Nonetheless, this could be confirmed as a true solution using mapCC (global mapCC 0.47) and further underlined as such using *ModelCraft* (Bond & Cowtan, 2022 ▸) to automatically rebuild the structure. *ModelCraft* was able to improve the model completeness from 35.7% to 77.1% and to improve the refinement scores (*R* factor 0.328, *R*_free_ 0.394). This example also demonstrates where the PAE approach can struggle due to a lack of distinguishable structural domains in the PAE/EPE plot (Fig. 6 ▸*c*).

### 3.3. Map–model binary-classifier results

When adapting *Slice’N’Dice* EM, we encountered an issue with classifying a properly fitted model. In MR, output scores from programs can confidently indicate whether a model has been correctly positioned, as discussed in Section 2.3.1. However, in EM cases, while there are validation scores available, they could not be used to reliably determine placement success. This prompted the development of a logistic regression binary classifier for *Slice’N’Dice*, which evaluates the fitting positions of models based on several map–model scores (see Section 2). The classifier produces a probability score between 0 and 1, with values closer to 1 indicating greater agreement between the model and the map. A cutoff value of 0.5 is set, with all values that are greater being given a success classification.

**Figure 7:**
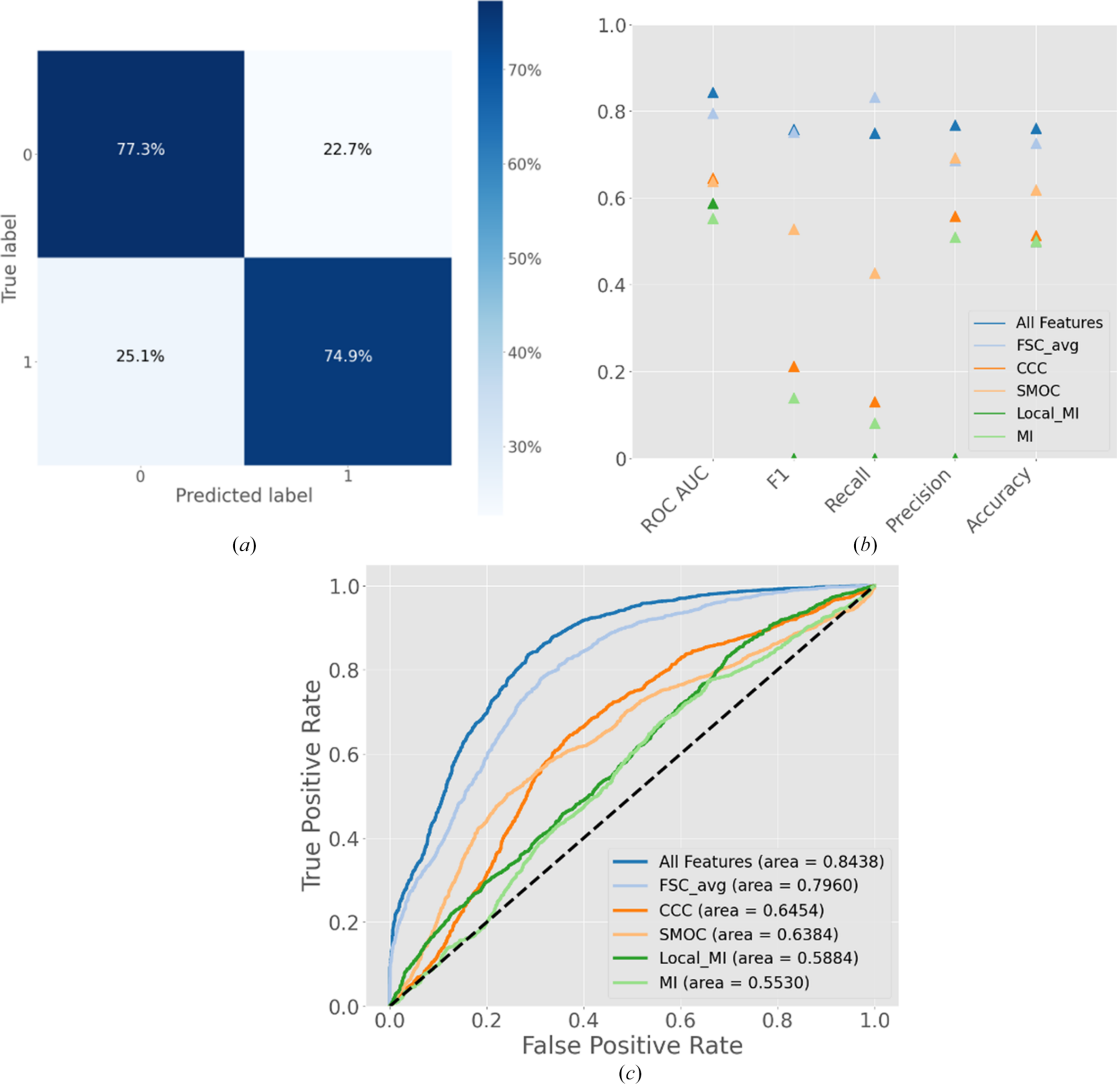
Classifier validation plots comparing an all-features classifier against classifiers trained on single features. (*a*) Confusion matrix for the entire data set (4438 rows of input features). (*b*) Classifier validation metrics for each classifier trained on single metrics against the classifier trained on all metrics comparing overall ROC AUC, F1 score, recall, precision and accuracy. (*c*) Receiver operating characteristic (ROC) curve for each classifier. ROC AUC, receiver operating characteristic area under the curve.

To assess the effect of the multiple feature inputs, classifiers trained on single features were compared against the classifier trained with all features. The classifier trained on all features outperformed the other classifiers, indicating a synergistic effect. Across all metrics, the ‘All features’ classifier showed the best discriminatory power to classify the success and failure classes. From Fig. 7 ▸, it is apparent that the metrics of the FSC average classifier were greater than its counterparts and almost close to the ‘All features’ classifier, yet it was surpassed on every metric except recall. A high recall and low precision indicate that the FSC classifier is producing more false positives than the ‘All features’ classifier (Fig. 7 ▸*b*). Such false positives could disproportionately negatively impact the overall success of *Slice’N’Dice*: incorrectly placed slices could block regions of the map and prevent the fitting of a potentially correct placement of another slice. To further assess the effect of multiple features, single features were systematically dropped, *i.e.* an ablation study was conducted. Interestingly, the choice to include ‘Resolution’ as an input feature caused a marginal decrease in performance: an ROC AUC of 0.830 with resolution and 0.844 without resolution. After removing resolution as an input feature, each further feature that was dropped decreased the overall performance of the model. Taken together, these observations clearly illustrate the synergistic effect of the input features.

**Figure 8:**
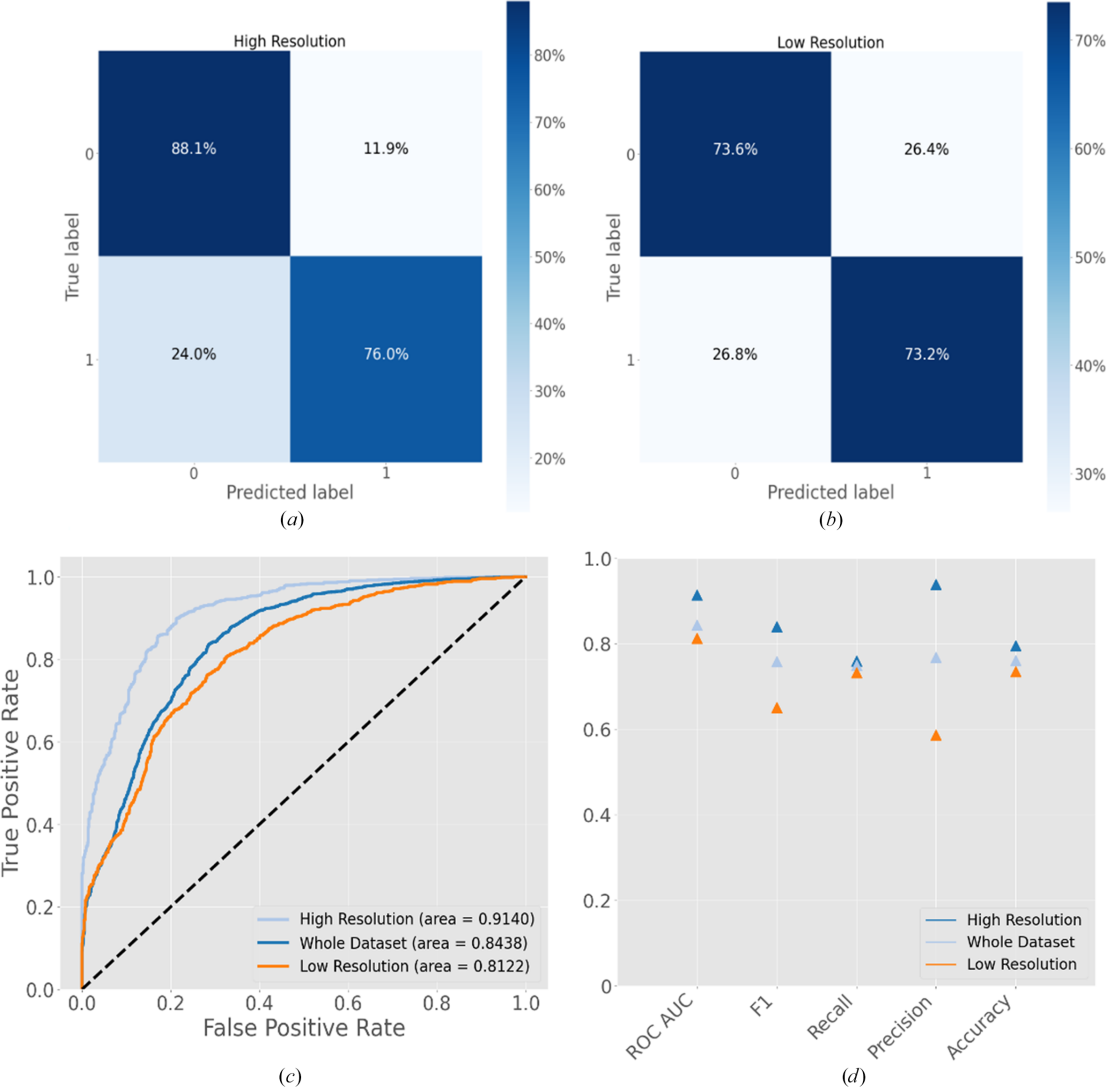
Classifier validation plots. (*a*) Confusion matrix for a subset of the complete testing data set classified as ‘high’ resolution (≤4 Å). (*b*) Confusion matrix for a subset of the complete training data set classified as ‘low’ resolution (>4 Å). (*c*) Receiver operating characteristic (ROC) curve for the resolution groups. (*d*) Classifier validation metrics for each resolution group, comparing overall ROC AUC, F1 score, recall, precision and accuracy. ROC AUC, receiver operating characteristic area under the curve.

The performance was then compared at high resolution (≤4 Å) or low resolution (>4 Å). Figs. 8 ▸(*a*) and 8 ▸(*b*) show the confusion matrices from ‘high’ and ‘low’ resolution subsets of the testing data set, respectively. The proportion of the data that are false negatives remains fairly consistent between the two, although the proportion of false positives is higher in the low-resolution subset (26.4%) than the higher resolution subset (11.9%), presumably indicating the increased difficulty in assessing placements at low resolutions. Nonetheless, *Slice’N’Dice* still produces good results in the lower resolution range, as the examples below show.

### 3.4. EM examples

#### 3.4.1. Example 1: PDB entry 7ymt (EMDB entry EMD-33942)

**Figure 9:**
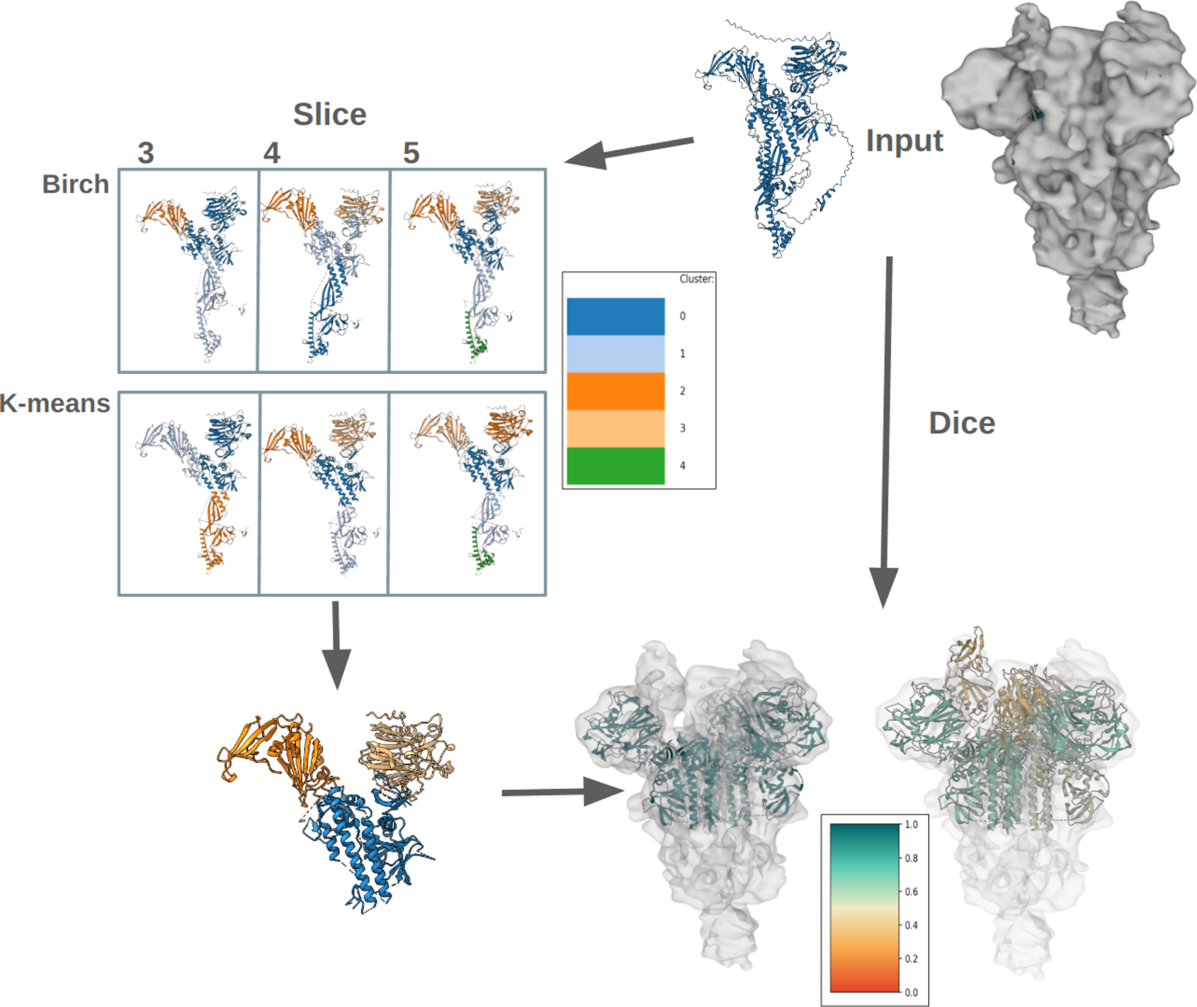
Pipeline following *Slice’N’Dice*, made possible by the *CCP-EM* software suite (Burnley *et al.*, 2017 ▸) GUI *Doppio* (Burnley *et al.*, 2023 ▸). In the suite, *Slice’N’Dice* is split into three jobs: *Slice*, *Dice* and *Slice’N’Dice*. The model is a *ColabFold* model of the SARS-CoV-2 spike protein PDB entry 7ymt generated using *ColabFold* (Mirdita *et al.*, 2022 ▸) with pLDDT scores stored in the *B* factor column of the PDB file. Residues under a certain threshold (70; the default and the value used in generation of the figure) are trimmed and the remaining residues undergo the slicing process. In this figure, two separate slice jobs were performed using *BIRCH* and *k*-means clustering (Pedregosa *et al.*, 2011 ▸). (n.b. the colours for each slice are inconsistent between jobs and split numbers. A colour key is supplied with each run to indicate which filename links to each colour.) For the purpose of this exercise, *k*-means split 4 was used without slice 1 as this slice contains extraneous features. The *Dice* job is run next using the input models from the *Slice* job. An input map is used as the template for the map fitting. In this example, this is a 6.55 Å resolution reconstruction of the spike protein from the EMDB (EMD-33942). After the map fitting, two output windows are displayed in *Doppio*, one containing the models that passed the classifier check and a second output of the combined ‘passed’ models and any remaining models. These are coloured according to the confidence score of the classifier, an extra output for the *Dice* job.

EMDB entry EMD-33942 is a map of the MERS-CoV spike protein, with a reported global resolution of 6.55 Å (Gecht *et al.*, 2022 ▸). The solved structure has PDB entry 7ymt. The map represents a protein trimer of the spike glycoprotein with a pseudo-symmetry of *c*3. Fig. 9 ▸ demonstrates the use of *Slice’N’Dice* by dividing the task into *Slice* and *Dice*. *Slice*, the model-splitting step, was run using two different clustering methods (*BIRCH* and *k*-means; Fig. 1 ▸). The range of slices was set to between three and five. The four slices of the *ColabFold* monomer model made from *k*-means were selected to go forward into the *Dice* job, our automated map-fitting pipeline, but one was disconsidered because it was fragmented after pLDDT trimming and did not resemble a clear domain (Fig. 7 ▸). *Slice’N’Dice* successfully managed to place six domains (from a total of 12) confidently into the map with a global cross-correlation (CC) score of 0.84 (Table 3 ▸).

#### 3.4.2. Example 2: PDB entry 8gtd (EMDB entry EMD-34250)

**Figure 10:**
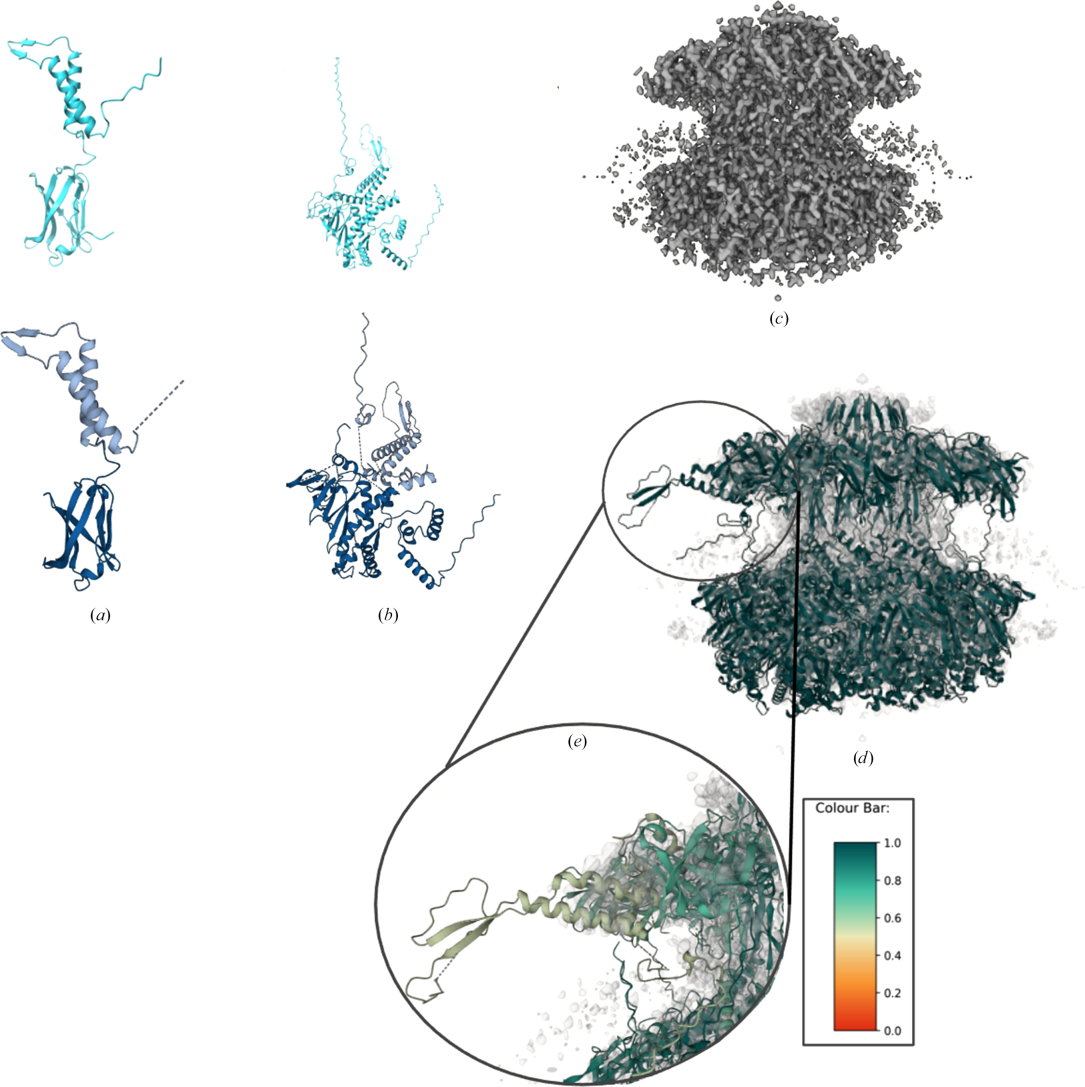
Results output from a *Slice’N’Dice* job. (*a*) PDB entry 8gtd chains *A* (*a*) and *B* (*b*) were generated using *ColabFold* (Mirdita *et al.*, 2022 ▸). Cyan models represent the unsplit *ColabFold* models. Both were split into two slices using *BIRCH* clustering, which is the default in *Slice’N’Dice*. The colouring to represent each cluster is shown in (*e*). The slice on chain *A* segmented the model suitably; however, chain *B* was not segmented into domains as clearly, although this did not significantly impact the overall results. *Slice’N’Dice* run alongside the input map file EMDB entry EMD-34250 (*c*) managed to place 45 out of 48 placements into the correct locations (*d*). However, one false positive passed the classifier check (*e*) and was placed in the head-to-tail joining protein instead in the portal protein. Utilizing the probability scores generated by the pipeline, it is evident that the placement has a lower score (∼0.52) than its nearest counterpart (∼0.78) and should be treated as a less confident result by the end user. The probability score is between 0 and 1.

EMDB entry EMD-34250 is a map of a marine siphophage protein, with a global resolution reported to be 4.7 Å (Huang *et al.*, 2023 ▸). The density file comprises four regions: the portal–adaptor complex, which consists of two of the four regions, the terminator and the tail tube. The solved structure (PDB entry 8gtd) for the portal–adaptor complex consists of a C12 formation of two distinct protein chains: the portal protein and the head-to-tail joining protein. As PDB entry 8gtd only corresponded to the portal–adapter complex, the terminator and tail tube were manually removed from the map using *ChimeraX Segger* (Pintilie *et al.*, 2010 ▸). In the original paper, the solved structure was generated using the *trRosetta* server (Du *et al.*, 2021 ▸) and the placements were manually fitted into a map file using *UCSF ChimeraX* (Pettersen *et al.*, 2021 ▸). A target such as this with many chains is an ideal candidate for automated model preparation and map fitting. Each chain was sliced twice using the *BIRCH* clustering algorithm in *Slice’N’Dice*. Splitting each chain into two domains allowed *Slice’N’Dice* to more accurately place these models by accounting for inter-domain orientation issues that had arisen during modelling. The final CC was 0.48 and out of a possible 48 slices, *Slice’N’Dice* was successful in placing 45 with one false positive (Fig. 10 ▸).

#### 3.4.3. Example 3: PDB entry 8bx5 (EMDB entry EMD-16308)

EMDB entry EMD-16308 is a map of a nicotinic acetyl­choline receptor from *Alvinella pompejana* (De Gieter *et al.*, 2023 ▸) with a global resolution reported as 4.2 Å. The density file represents a homopentamer.

**Figure 11:**
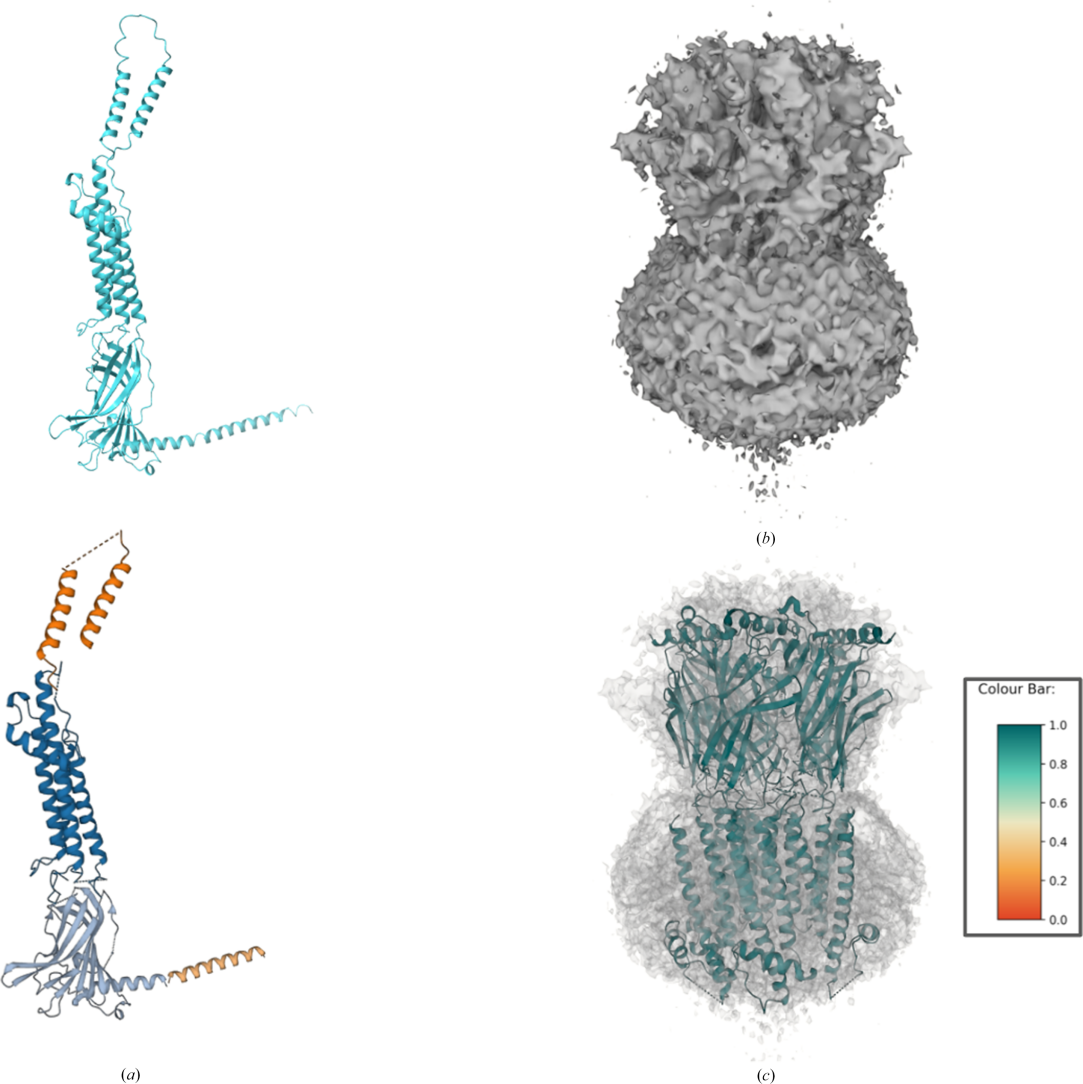
PDB entry 8bx5 was generated using *ColabFold* (Mirdita *et al.*, 2022 ▸). (*a*) Cyan models represent the unsplit *ColabFold* models. Chain *A* was split into four slices using *BIRCH* clustering, which is the default in *Slice’N’Dice*. *Slice’N’Dice* segmented the chain *A* model suitably. (*b*) The map input into *Slice’N’Dice*. (*c*) *Slice’N’Dice* successfully managed to place the majority of the map with high confidence (∼0.97–0.99 per placement).

The *ColabFold* (Mirdita *et al.*, 2022 ▸) model generated was a close match to the deposited model (PDB entry 8bx5) although residues 308–412 were not visible in the map (De Gieter *et al.*, 2023 ▸). *Slice’N’Dice* was run with the default *BIRCH* clustering method and sliced the model into four. Among the four slices, the two largest (Fig. 11 ▸) were fitted into the map successfully, filling most of the available map. The success can be witnessed by the dark green result model (Fig. 11 ▸*c*), denoting a high confidence score (∼0.99 per placement; Fig. 11 ▸*c*). Additionally, the two slices corresponding to residues 308–412 were successfully rejected (∼0.37 per placement). This showcases the ability of *Slice’N’Dice* to differentiate between models that are present and absent in the density. Overall, *Slice’N’Dice* fitted six out of nine possible placements and the final CC was 0.71.

## 4. Graphical user interfaces for *Slice’N’Dice*

Access to all of the controlling parameters of the program can be made from the command line. *Slice’N’Dice* has also been integrated into several graphical user interfaces (GUIs) provided by the *CCP*4 and *CCP-EM* suites.

### 4.1. *Moorhen* interface

**Figure 12:**
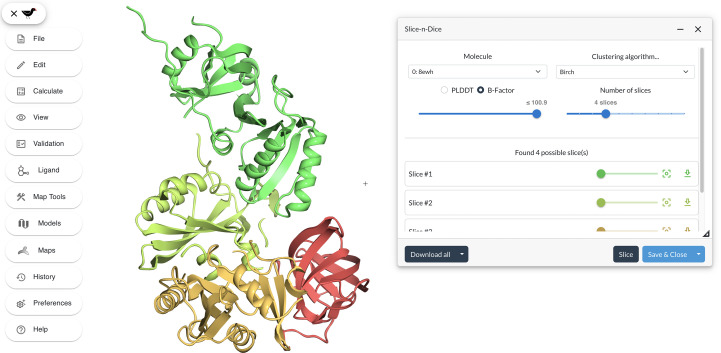
The *Slice’N’Dice* interface in *Moorhen* was used to ‘slice’ PDB entry 8ewh into four distinct slices using the *BIRCH* algorithm. No *B* factor trimming was applied before clustering.

*Moorhen* (https://moorhen.org/) is a React-based web-enabled molecular-graphics interface to the *Coot* interactive model-building application (Emsley *et al.*, 2010 ▸). An interface for *Slice’N’Dice* has been added into *Moorhen* (Fig. 12 ▸). To facilitate the use of the clustering algorithms in a web environment, the clustering methods used in *Slice’N’Dice* were implemented using the C++ programming language. The resulting library was then compiled into WebAssembly using Emscripten (Zakai, 2011 ▸) and a custom React-based interface was created to let users execute the following clustering algorithms: *BIRCH* (Zhang *et al.*, 1997 ▸), agglomerative (Murtagh & Contreras, 2012 ▸), *k*-means (Lloyd, 1982 ▸) and PAE clustering (Oeffner *et al.*, 2022 ▸). The resulting plugin is available in *Moorhen* and can be used to ‘slice’ molecules into distinct domains. Additionally, prior to this clustering, users can define a threshold by which residues in the input model can be trimmed based on their *B* factor or pLDDT values. This residue trimming is performed using the *GEMMI* library (Wojdyr, 2022 ▸), which was also compiled using Emscripten. *Moorhen* is integrated into *CCP*4 Cloud (Krissinel *et al.*, 2022 ▸) and *Doppio* (Burnley *et al.*, 2023 ▸) and will soon be available through *CCP*4*i*2 (Potterton *et al.*, 2018 ▸).

An advantage of the more interactive, graphically driven approach implemented in *Moorhen* is that it allows a user to tweak the trimming threshold visually. This can subsequently influence the clustering of the atoms to produce a different splitting of the model depending on the trimming threshold that has been selected. It also allows the user to see the effect of choosing different numbers of slices, helping to isolate the optimum number of slices required for success in MR. In both *CCP*4 Cloud and *Doppio*, sliced models created using *Moorhen* are automatically saved and made available to any subsequent MR or map-fitting application.

### 4.2. *CCP*4 and *CCP-EM* interfaces

**Figure 13:**
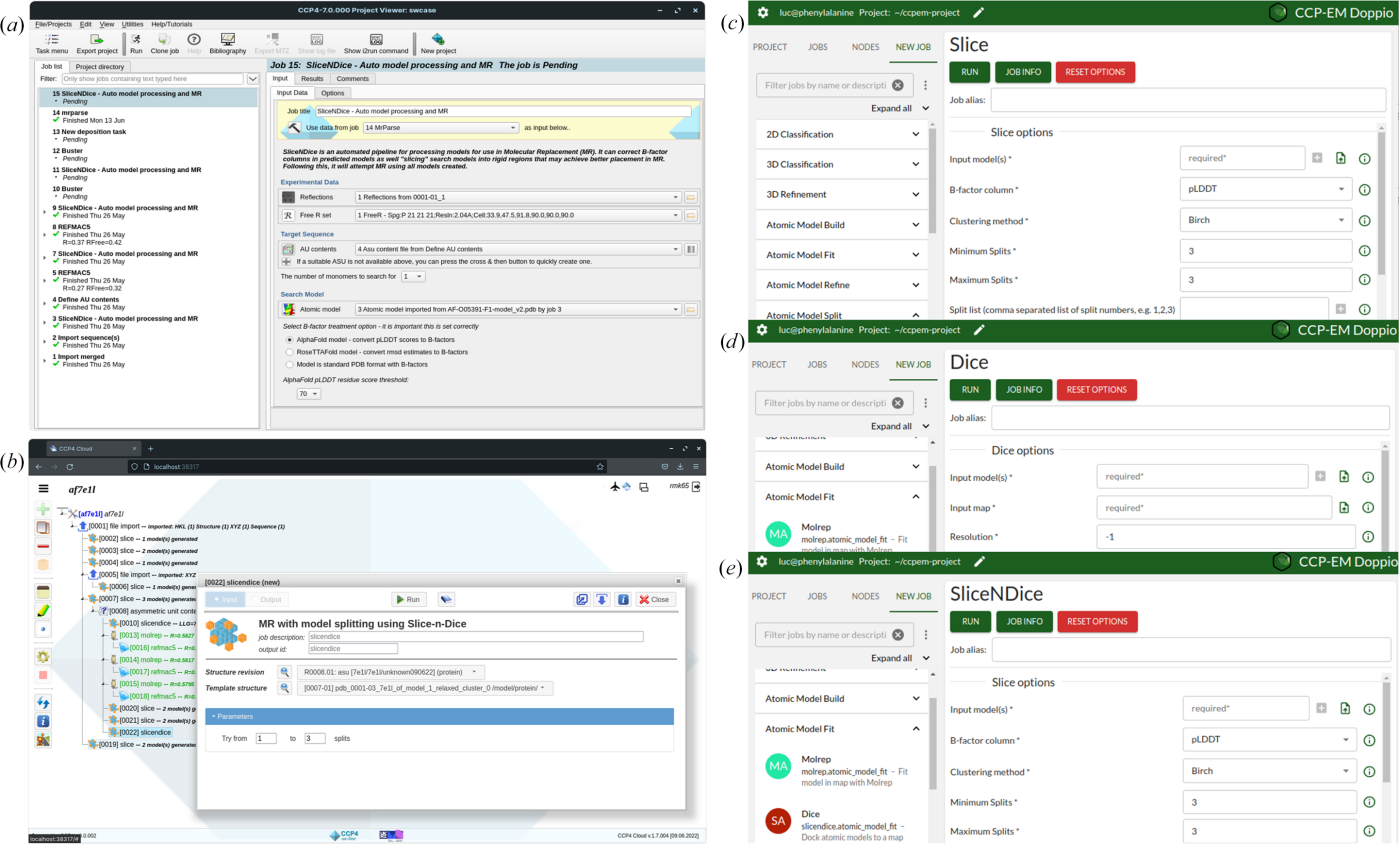
Screenshots of the *CCP*4 and *CCP-EM* GUIs. (*a*) *CCP*4*i*2 interface page for *Slice’N’Dice*. (*b*) *CCP*4 Cloud interface page for *Slice’N’Dice*. *Doppio* (Burnley *et al.*, 2023 ▸) interface cropped pages for the jobs (*c*) *Slice*, (*d*) *Dice* and (*e*) *Slice’N’Dice*.

*Slice’N’Dice* is available through both the *CCP*4 (Agirre *et al.*, 2023 ▸) and *CCP-EM* software suites. It has been incorporated into three *CCP4*/*CCP-EM* graphical user interfaces (GUIs): *CCP*4*i*2 (Potterton *et al.*, 2018 ▸), *CCP*4 Cloud and *Doppio* (Fig. 13 ▸). These provide interfaces for slicing models (*Slice*), for automated map fitting (*Dice*) and for model slicing followed by automated MR or automated map fitting (*Slice’N’Dice*). For MX use, *Slice’N’Dice* on the command line allows users to select more runtime options, but the *CCP*4 interfaces provide a quick and easy way to run *Slice’N’Dice*. For EM use, all functionality is available through the *Doppio* interface.

## 5. Discussion and conclusions

*Slice’N’Dice* offers an easy and automated means to address cases where the conformation of a structure prediction, especially in terms of inter-domain orientations, differs significantly from that of the target.

For crystallographers, *Slice’N’Dice* can significantly improve the chance of MR success. Here, we showed that clustering algorithms can be used to identify distinct structural units within a model that may not be immediately obvious when visually inspecting the structure. Currently, the default clustering algorithm used by *Slice’N’Dice* is *BIRCH* (Zhang *et al.*, 1996 ▸). While *BIRCH* has performed well throughout the development stage of *Slice’N’Dice*, alternatives will be benchmarked in the future, potentially including clustering methods such as *SWORD*2 (Cretin *et al.*, 2022 ▸), *DCI* (Kumar *et al.*, 2022 ▸), *Merizo* (Lau *et al.*, 2023 ▸) and *Chainsaw* (Wells *et al.*, 2024 ▸), as well as alternative clustering methods provided by *scikit-learn*. We will also look at the combination of clustering methods in a consensus strategy. *DCI*, using predicted motions for definition of structural units, might be particularly relevant given that the dynamic properties of multi-domain proteins underlie some of the difficulties that *Slice’N’Dice* is designed to address. We are also aware that clustering in combination with the removal of low-confidence residues can occasionally leave disconnected fragments (Fig. 6 ▸*b*): recognizing that these might impact on the packing of solutions, we will explore methods to identify and eliminate these.

For cryoEM practitioners, *Slice’N’Dice* EM offers an automatic solution for map fitting and assessing model placements within a single pipeline. The map–model binary classifier generally differentiates well between correct and incorrect fits, although the EM example PDB entry 8gtd illustrates a false-positive placement that was included (Section 3.2.1). In the *Doppio* interface, individual placed slices are coloured by probability score, so that false positives are often visually apparent as they typically have lower scores than the other correct placements. However, the ultimate goal must be to reduce false positives/negatives. To make further improvements, a larger training data set is being created with the aim of enhancing the performance of the classifier with challenging cases such as small single α-helical slices. When developing the classifier, an assumption was that the resolution would have been useful information for the classifier. However, the results proved otherwise, and the classifier performance improved when resolution was not given as an input variable. There are at least two possible reasons for this observation. Firstly, at lower resolution there could be more errors in the deposited structures used as reference structures to generate the target variables. Alternatively, it could be that the global resolution was misleading in some cases, *i.e.* that providing global resolution as input does not provide accurate information about the local resolution surrounding the placement. It was observed that the classifier discriminates better when working with maps of a higher resolution than lower resolution (Fig. 8 ▸). A future development point could be to calculate the average local resolution of the area around a docked slice and provide this information to an improved classifier. Another point could be to explore the usefulness of *Slice’N’Dice* EM for cryo-electron tomography (cryoET). In this manuscript, we focused on single-particle analysis for the cryoEM examples, but due to the ability of *Slice’N’Dice* to perform well at lower resolutions (>4 Å; Fig. 8 ▸*b*) it will be useful for automated model building with subtomogram averages. Finally, we will also explore the use of *em_placement* and *emplace_local* (Millán *et al.*, 2023 ▸) as alternative map-fitting methods for cryoEM and cryoET.

As we were writing this manuscript, *AlphaFold*3 was released. Whilst *AlphaFold*3 is an improvement on *AlphaFold*2, it may still mis-predict relative domain conformations (Abramson *et al.*, 2024 ▸). *Slice’N’Dice* is compatible with *AlphaFold*3 output models and the accompanying expected position error information (comparable to the PAEs of *AlphaFold*2), and should therefore remain useful for MR/map fitting.

## 6. Related literature

The following references are cited in the supporting information for this article: Brown *et al.* (2015 ▸), Farabella *et al.* (2015 ▸), Fontana *et al.* (2022 ▸), van Heel & Schatz (2005 ▸), Joseph *et al.* (2017 ▸), wwwPDB Consortium (2024 ▸) and Yamashita *et al.* (2021 ▸).

## Supplementary Material

Supplementary information for cryo-EM part of paper including a description of machine-learning implementation. DOI: 10.1107/S2059798325001251/qe5007sup1.pdf

## Figures and Tables

**Table 1:** Hyperparameter search using RandomizedSearchCV The search space comprises the range of values for the search to combine, along with the selected values that, when combined, yielded the best score. The model was changed as the perceptron model does not produce probability values. Instead, log (logistic regression) was selected.

Hyperparameter	Search space	Selected value
Alpha	[0.0001, 0.001, 0.01, 0.1, 1, 10, 100]	0.001
Penalty	[l2, l1, elasticnet, none]	l2
Learning Rate	[optimal, invscaling, adaptive]	invscaling
Eta0 (the initial learning rate)	[0.01, 0.1, 1, 10, 100]	100
Loss (or choice of model)	[hinge, log, modified_huber, squared_hinge, perceptron]	perceptron (log)
Power_td	[0.1, 0.2, 0.5, 0.55, 0.9]	0.9
Validation Fraction	[0.1, 0.2, 0.3]	0.2
Epsilon	[0.1, 0.2, 0.3]	0.1

**Table 2:** Summary of the example results for MX Each case has a single copy in the asymmetric unit. For PDB entry 7oa7 the results using PAE *networkx* are shown in parentheses. The fraction of atoms accepted in the r.m.s.d. calculation are shown in square brackets. In the case of PDB entry 7b9c only three of the four slices were placed by *Phaser*. The predicted model also represented about 40% of the scattering content in the crystal structure. Nonetheless, the MR scores and the mapCC values clearly indicate the correct placement of the three slices found.

	PDB entry 7oa7	PDB entry 7rb4	PDB entry 7b9c
Resolution (Å)	1.45	2.19	2.4
No. of reflections	81269	28929	133845
Clustering method	*BIRCH* (PAE *networkx*)	*BIRCH*	*BIRCH*
No. of slices	2	3	4
R.m.s.d. of entire model to target before *Slice’N’Dice* (Å)	1.528 [0.84] (1.083 [0.74])	3.987 [0.86]	3.952 [0.53]
R.m.s.d.s of slices to target after *Slice’N’Dice* (Å)	0.505 [0.91], 0.550 [0.85] (1.213 [0.87], 0.488 [0.84])	0.528 [0.84], 0.757 [0.79], 0.9 [0.74]	0.466 [0.71], 0.616 [0.91], 0.797 [0.99], 1.038 [0.76]
Model completeness/scattering content (%)	91.5 (89.1)	100	37.5
*Phaser* LLG	1339 (641)	114	310
*Phaser* TFZ	22.5, 35.6 (20.3, 18.5)	6, 11.6, 13.2	13.0, 20.7, 27.4
*REFMACR* factor	0.41 (0.41)	0.44	0.48
*REFMAC* *R*_free_	0.41 (0.41)	0.47	0.51
Local mapCC	0.79 (0.79)	0.72	0.80
Global mapCC	0.7 (0.7)	0.59	0.47

**Table 3:** Summary of the EM example results

	PDB entry 7ymt	PDB entry 8gtd	PDB entry 8bx5
EMDB code	EMD-33942	EMD-34250	EMD-16308
Resolution (Å)	6.55	4.7	4.2
Clustering method	*k*-means	*BIRCH*	*BIRCH*
Slices placed	6/9	45/48	9/15
No. of slices	3	2, 2 (multi-chain input)	4
Solved structure CC	0.9317	0.7273	0.8633
*Slice’N’Dice* output CC	0.8436	0.4808	0.7126
