# Supplementary material for: Slice’N’Dice: maximizing the value of predicted models for structural biologists
Source: Acta Crystallogr D Struct Biol. 2025 Feb 20;81(Pt 3):105–21. doi: 10.1107/S2059798325001251 (PMC11883665; doi:10.1107/S2059798325001251)
Supplement: Supplementary file 1 [file d-81-00105-sup1.pdf]

STRUCTURAL
BIOLOGY

Volume 81 (2025)

Supporting information for article:

Slice'N'Dice: maximizing the value of predicted models for structural biologists

Adam J. Simpkin, Luc G. Elliot, Agnel Praveen Joseph, Tom Burnley, Kyle Stevenson, Filomeno Sánchez Rodríguez, Maria Fando, Eugene Krissinel, Stuart McNicholas, Daniel J. Rigden and Ronan M. Keegan

S1. EM supplementary information

S1.1. Training data generation

A total of 124 map files were selected from the EMDB. A random sampling of globally reported resolutions in the range of 2.3Å to 13.0Å was performed, with a requirement of having a corresponding solved structure. This approach ensures a ground truth "reference" model for training purposes. Deposited structures are prone to errors; however, the target variable generation is split into positive and negative classes based on a cutoff. This cutoff is more generous than a continuous score, and the impact of local errors is minimised if the global comparison is good. To increase exposure to lower resolution maps, a collection of higher resolution maps (< 4Å) maps were low pass-filtered to different resolution bins: 6Å, 8Å, 10Å and 12Å. This was used alongside experimental, lower resolution maps.

In order to learn patterns and be used beyond training datasets, a dataset was curated of different map-model fitting scores with supplementary information. As a predicted typical use-case of *Slice'N'Dice* will be using *ab-initio* protein models, predicted models were generated to create a training dataset. For each chain in the solved structure a *ColabFold*, running *AlphaFold2* (Mirdita et al. 2022), was used to generate each chain ranging in size from <100 residues to >1000 residues. (December 2022 - January 2023) Each of these predicted models was split up using the Birch clustering algorithm according to Table 1, which were then docked into the map files.

Table S1 Amount of 'splitting' the training dataset of *AlphaFold2* models underwent alongside the approximate size ranges. AA = Amino acid.

Amino acid (AA) length classes	Slice range	Slice size
<100AAs	1-2	LOWEST – ~100 AAs
101AAs – 450AAs	2-3	~33AAs – ~225AAs
451AAs – 700AAs	2-4	~113AAs – ~350AAs
701AAs – 1000AAs	3-5	~140AAs – ~333AAs
>1000AAs	4-6	~167AAs – Highest

Table S2 Different map-model scores*.

Before scoring, a simulated volume is generated from the input model. The x and y variables represent density values of the voxels of the probe (simulated map from model) and target maps (comparison volume, typically experimental data) respectively. * and resolution. **Feature importance of a logistic regression model is represented as coefficient magnitude, a larger value indicates a larger magnitude of importance between the input variables. FSC = Fourier Shell Correlation, MI = Mutual Information CC = Cross Correlation, EMDB = Electron Microscopy Database, SMOC = Segment Based Manders' Overlap Coefficient.

Classifier input features (Map-model scores*)	Calculated
**CC**	A Pearson's product moment correlation coefficient calculation. CC measures a linear correlation between the probe map and the target map (Joseph et al. 2017). $$CC(x, y) = \frac{\Sigma(x-\bar{x})(y-\bar{y})}{\sqrt{\Sigma(x-\bar{x})^2} * \sqrt{\Sigma(y-\bar{y})^2}}$$
**FSC Average**	FSC is a quantitative measure of the normalised cross-correlation between the probe map and target map in Fourier space (Equation 1: (van Heel and Schatz 2005)). To calculate the FSC Average, the FSC is averaged across the number of shells. (van Heel and Schatz 2005; Brown et al. 2015). Where N is the number of shells and FSC_i is the FSC correlation coefficient at the corresponding shell. $$FSC_{average} = \frac{\sum_{i=1}^{N_{shell}} N_i FSC_i}{\sum_{i=1}^{N_{shell}} N_i}$$
**MI**	MI is a measure of relative entropy (probability distributions) between the joint distribution p(x,y) and the product distribution p(x)p(y). p(x) and p(y) denote the percentages of voxels that have density values equal to x and y, respectively (Farabella et al. 2015). $$MI(x,y) = \sum_{x\in X}\sum_{y\in Y} p(x,y) log(\frac{p(x,y)}{p(x)p(y)})$$
**Local MI**	Local MI is calculated similarly to MI but is broken down into bins (k) and OVR_{XY} is the number of voxels within the overlap region (Joseph et al. 2017): $$k = [1 + log_2(OVR_{xy})]$$
**Overlap scores: map and model**	The overlap score represents the ratio of overlapping voxels OVR_{xy} against the total number of voxels within the defined contour (Joseph et al. 2017). $$OVR_{Model} = \frac{OVR_{xy}}{n_x}$$ $$OVR_{Map} = \frac{OVR_{xy}}{n_y}$$
**SMOC Average**	Product moment coefficient without the deviation from the mean. Giving a per residue score instead of a global score. An average is taken for input into the classifier (Joseph et al. 2017). $$SMOC(x, y) = \frac{\Sigma(xy)}{\sqrt{\Sigma(x)^2} * \sqrt{\Sigma(y)^2}}$$

Map-model scores quantify how well a model has been docked into a map file. Map-model FSC scores are a way of assessing a placement into a map-file, e.g *Refmac5* FSC average for monitoring the refinement of a model into a map (Yamashita et al. 2021). Another score which is widely used in the Cryo-EM field is Cross-Correlation (CC): this, however, is influenced by the shape and size of density distributions. The grid size of the map file and the chosen contour level can affect CC score (Joseph et al. 2017). This makes it difficult to pick a threshold value of a successful placement. To address this issue, a machine learning classifier was developed that takes various map-model scores as input and attempts to deduct whether a successful placement has been made. See table 3.1.2 for a full list of input variables. Many of these scores compare the real-space voxel positions between a simulated density representation of an atomic model and the map file. These scores took a contour level, taken from the author recommended tab from the map file's EMDB page (wwPDB Consortium 2024). to discern which voxels are used in the calculation.

For each docked model, the map-model scores were collected, see table 2 To train and test the classifier against the target variable on whether the model has been placed sufficiently or not, an r.m.s.d-based score was used alongside the map-model scores as input features. The map-model scores in Supplementary Table 1 do not represent the input features into the classifier. During feature selection, features were selectively removed if it increased model performance.

S1.2. Target variable generation

To train and test the classifier against the target variable on whether the model has been placed sufficiently or not, an r.m.s.d-based score was used alongside the map-model scores as input features.

To begin, each residue on the docked model is iterated over, with the α-carbon atom position used as a gridpoint in 3D-space. A search volume of 5Å is created around each residue to find all residues of the reference model. The residue on the reference model with the smallest relative distance is then paired with the current docked model residue. This does not necessarily pair up corresponding residues. When generating map-model scores, residue identification is not taken into account. If residue information were to be considered, it could lead to an inflated r.m.s.d-based score in regions where there is structural homology but distinct residue sequences. This could potentially confuse the classifier and result in incorrect classifications. In cases where there are structural homologues, *Slice'N'Dice* can be used as a template for homology modelling, similar to what was accomplished by (Fontana et al. 2022) when building a nucleoporin to solve a larger nuclear pore complex.

This process is repeated for each residue iteration. If a residue on the reference structure is already linked to another residue on the docked model, the distances are compared, and the pairing with the lowest distance is selected. These iterations continue until the number of paired residues no longer changes. An r.m.s.d. calculation is then performed between these pairs. If some residues on the docked model have not been paired, a coverage penalty is applied to the r.m.s.d. score. The r.m.s.d. value is divided by the coverage, for example, if only 20% of the docked model found a nearby pair, the r.m.s.d. of the paired residues would be divided by 0.2 or multiplied by five.

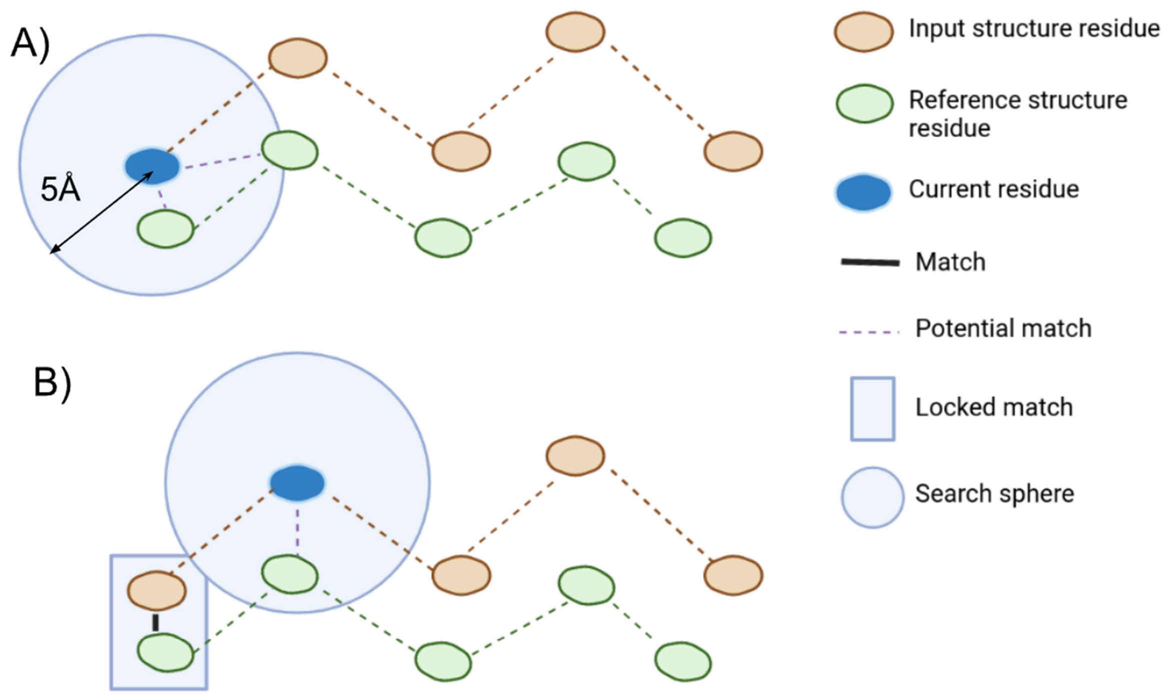

Figure S1 A 2-D visual representation of the process of generating pairs before the r.m.s.d-calculation. A and B represent the same docked model (orange) and reference model (green) at different iterations in order. The blue residue represents the residue that currently has the search volume surrounding it. In iteration A), the docked model residue matches two potential residues on the reference model. In iteration B), only one residue matches with the reference model, and the previous residue is locked in a pair as it was the closest of the two potential matches. Note: the distances between the residues are not to scale and are merely to represent a bond.

S2. EMDB Correlation Coefficient Table

Table S3 The cross correlation (CC) of ~14K EMDB deposited CryoEM density reconstructions (maps) (wwPDB Consortium 2024) and their corresponding protein models.

The data is split into quartiles (Q1,Q2,Q3) at different resolution ranges. cc_mask refers to a mask around the corresponding model and the parts of the map within that mask were used in the score calculation. The entries were selected based on having half maps available. Half-maps, however, were not used for the CC score calculation.

Metric	Resolution (Å)	No. of depositions	Q1	Q2 (Median)	Q3
**cc_mask**	0.0-2.5	970	0.454	0.526	0.6085
	2.5-3.5	7116	0.443	0.5	0.584
	3.5-4.5	3471	0.414	0.49	0.59
	4.5-6.5	537	0.408	0.507	0.62375
	>6.5	394	0.4345	0.559	0.694

References

Brown, Alan, Fei Long, Robert A. Nicholls, Jaan Toots, Paul Emsley, and Garib Murshudov. 2015. “Tools for Macromolecular Model Building and Refinement into Electron Cryo-Microscopy Reconstructions.” *Acta Crystallographica. Section D, Biological Crystallography* 71 (Pt 1): 136–53.

Farabella, Irene, Daven Vasishtan, Agnel Praveen Joseph, Arun Prasad Pandurangan, Harpal Sahota, and Maya Topf. 2015. “TEMPy: A Python Library for Assessment of Three-Dimensional Electron Microscopy Density Fits.” *Journal of Applied Crystallography* 48 (Pt 4): 1314–23.

Fontana, Pietro, Ying Dong, Xiong Pi, Alexander B. Tong, Corey W. Hecksel, Longfei Wang, Tian-Min Fu, Carlos Bustamante, and Hao Wu. 2022. “Structure of Cytoplasmic Ring of Nuclear Pore Complex by Integrative Cryo-EM and AlphaFold.” *Science* 376 (6598): eabm9326.

Heel, Marin van, and Michael Schatz. 2005. “Fourier Shell Correlation Threshold Criteria.” *Journal of Structural Biology* 151 (3): 250–62.

Joseph, Agnel Praveen, Ingvar Lagerstedt, Ardan Patwardhan, Maya Topf, and Martyn Winn. 2017. “Improved Metrics for Comparing Structures of Macromolecular Assemblies Determined by 3D Electron-Microscopy.” *Journal of Structural Biology* 199 (1): 12–26.

Mirdita, Milot, Konstantin Schütze, Yoshitaka Moriwaki, Lim Heo, Sergey Ovchinnikov, and Martin Steinegger. 2022. “ColabFold: Making Protein Folding Accessible to All.” *Nature Methods* 19 (6): 679–82.

Pedregosa, Fabian, Gaël Varoquaux, Alexandre Gramfort, Vincent Michel, Bertrand Thirion, Olivier Grisel, Mathieu Blondel, et al. 2011. “Scikit-Learn: Machine Learning in Python.” *Journal of Machine Learning Research: JMLR* 12 (85): 2825–30.

wwPDB Consortium. 2024. “EMDB-the Electron Microscopy Data Bank.” *Nucleic Acids Research* 52 (D1): D456–65.

Yamashita, K., C. M. Palmer, T. Burnley, and G. N. Murshudov. 2021. “Cryo-EM Single-Particle Structure Refinement and Map Calculation Using Servalcat.” *Acta Crystallographica Section D: Structural Biology* 77 (10): 1282–91.
